# Editorial

**Published:** 1896-10

**Authors:** 


### Editorial.

"In a recent issue of a magazine published in Atlanta appears a little squib, intended, we presume, as a modern Joe Miller jest, though lacking in two of the three essentials which characterize the jokes, *bon mots*, etc., of the first edition of Wits' Vade-Mecum, namely, wit and humor; obscenity, which is the third, doubtless describes our esteemed contemporary's melancholy attempt.

"The joke is based upon an alleged story of a French peasant woman using a lemon as a pessary for twenty-two years. We will not repeat it. It is too dismal!"—*Moody's Magazine of Medicine*, Sept., 1896.

THE "TOO DISMAL" JOKE.

"The story is going the rounds of a French woman who used a lemon as a pessary for twenty-two years, changing it every two or three months. From what is known of French peasant women, the wonder is that the lemon should have remained in such a locality for so long without having been squeezed."—*Southern Medical Record*, August, 1896.

We had decided to adopt a sort of *laissez passer* policy in regard to expression of our opinion of this newcomer from Edom, *Moody's Magazine of Medicine*, but having been unceremoniously dragged in by the heels to answer for a serious charge, to remain quiet would seem an acknowledgment of guilt.

Whether or not the charge of obscenity is sustained we leave to our readers to determine. The "too dismal" (to be repeated but not to be alluded to) joke was an attempt to cast out of the current a stale item that had vexed the eyes in nearly every exchange that came to our table. It was apparently effective in accomplishing this purpose.

But whether true or not true, the charge of obscenity coming from a publication which printed as a frontispiece of its first number a wretchedly executed copy of one of Bouguereau's most meretricious nudities, if it were not provoking would be positively amusing. Does such a gratuitous piece of obscenity, that might well grace the walls of a Collins street

*maison de joie*, especially "fit" this magazine for the home? If so, the natural query is, for what sort of a home is it intended? The charge of obscenity from such a turbid source as this is difficult to bear with philsophical equanimity.

But inconsistence, one of the commonest of moral obliquities, is one of the least of the failings of this most surprising publication. Its miserable travesty of art, in which only the crudest of obscenity is apparent, even leans to virtue's side when compared with the weird and ghastly rot of its so-called editorial and literary departments. In the general inclusiveness of the term "literature" it is possible that the following gem taken from the Woman's department may have place, but it is more fitting to be regarded as an entomological curiosity to be pinned to a cork for preservation.

"The train is starting,
O God, the parting
Glance of your eye,
Good-bye, fair one, good-bye."

In the midst of these tender farewells, the following catastrophe overtook the poet:

"Just time for a look;
How I longed to linger;
My soul was *shook**
And my heart was slain
By a woman's look
And a moving train."

The editor, who is also the author of the foregoing, shows a power of cropping up again that is simply marvelous. *Ralcy redivivus*. After having been "shook" and even slain in a railroad accident, as we are informed above, he yet lives to pay a merited but tardy tribute to Jay Gould in words that the heirs of the lamented Jay should have carved upon his simple headstone, so that those coming after should know the sweet majestic character that was borne by this meek and unassuming gentleman. These things should be preserved in an enduring form, for posterity is apt to have an erroneous impression of some of our greatest men.

"The throne outlives the king; the coin, Tiberius."

Leaving this sad task of setting right before the world the character of a departed comrade, the versatile editor comes

*Italics our own.—S. M. R.

to the surface again as a champion of the much maligned Robert G. Ingersoll, and to show his infinite variety at once rushes into poetry again, this time in a prayer in which he poses as a flagellant and says that the only relief for his bleeding feet is to walk in a thorny place. Queer advice from a doctor.

To leave the bizarre "literary" features of this magazine for a broader theme the combination of medical articles with general literature is an singularly unfortunate one.

No one can be entirely at his ease before a mixed audience. A physician can not write freely and unreservedly upon subjects in his own rightful domain, which do not admit of discussion in polite society, when he has the consciousness that he will be read not only by an appreciative colleague, but also by the colleague's wife, and perhaps young daughters. Placed thus more or less directly in feminine company, he is constrained to change his tone from one of scientific candor to one of veiled allusion and artificiality—the effect of an assumption of "company manners." Withholding facts in deference to the proprieties is death to scientific inquiry. Nothing in science is base so long as it is scientifically considered, but there is a great deal in it that immediately becomes obscene when viewed from the standpoint of the general reader.

The promoters of this magazine have apparently overlooked this fact in setting forth their objects in their prospectus, or else have assumed that their readers can be hypnotized into being philosophers or fools, as the case requires. Unless their readers be thus susceptible, an article on sexual perversion *a la Krafft Ebing*, or a kindred subject, in a "popular" medical magazine would be regarded by the casual reader as salacious literature and read with the usual relish that attaches to such themes. And what is more, such an article is as likely to be read by the wife and daughter of the physician as one on dress-reform or what not, unless the discrete and cautious husband and father exercises a Muscovite censorship over the publication and blacks out the objectionable features before passing it on to the female members of his family.

Yet in a "medical" magazine, such as this, how are such things to be kept out, unless the contributors submit to gag rule?

A jumble of science and fiction reminds one of tragedy strutting with farce. Dignified science withdraws disdainfully from such company. A publication that contained an illustrated paper on radical cure of the piles joined cheek by jowl with the story of how Willie was lost in the Wild Woods, could not be regarded as the ideal one for the young of both sexes, although it would undoubtedly furnish very spicy reading for the family circle on rainy evenings.

There is a more serious side to this question than expediency.

Are we physicians to invite the public into our deliberations and consultations on the payment of twenty cents at any news-stand, and shall we beckon to any idle gazer in the crowd to approach nearer and witness our examinations and overhear our interrogations of a patient from whom we seek to gain a knowledge of the most secret workings of his or her inner life? The skeleton in the closet steps out nimbly to the call of the physician, but more carefully conceals himself at the approach of a curious, prying public. Success of a publication like *Moody's Magazine of Medicine*, in the attempt to popularize a knowledge of medicine by combining it with general literature as a sort of lubricant, would tend to do violence to the primitive conception of the duty of a physician to his patient. A paper on, for instance, a report of a series of cases of syphilis, in a popular publication such as this one desires to be, while not exactly a breech of confidence and trust on the part of the physician, because the names of the patients are not given, is perilously near it.

In the same vein, fancy reading a minute account of a case of midwifery in *Munsey's Magazine*, to make a long call for a comparison with Moody's!

Such incongruity is not only a grotesque dislocation but a degradation of medicine that should not be tolerated by any doctor who has regard for the dignity of his calling.

We can not but believe that the well-known and representative gentlemen who have so far contributed to this mongrel publication have not carefully considered these things, nor fully realized that in submitting their matter for publication in this would-be "popular" magazine, they are virtually writing to the general public, and might as well at once take a short cut and go to the newspapers. It is difficult to reconcile the well-known punctiliousness of some of these gentlemen with their apparent willingness to foregather with a "specialist" who was regurgitated from the State Association for flagrant violation of the code of ethics so recently that the episode is fresh in the minds of every one.

It is well to have some regard for the advisability of conservatism in these matters, particularly as concerns a publication which hangs, so to speak, by the gills in the matter of future policy. There is no dearth of legitimate medical journals in this country whose sole function it is to publish articles of merit on subjects in medicine which insures for the authors an intelligent, appreciative class of readers belonging to the medical profession, and not of laymen whose interest in medicine is too often prompted by a desire to satisfy a prurient curiosity and to whom a medical journal is comparable to a dime museum of anatomy.

In the end, it seems to us that some members of the most respectable medical element in this city are being cajoled by the subtleties of flattery into lending their patronage to further the material interests of some very slick gentry from the frozen North.

It sounds like slang to say that these medical gentlemen are being "worked." But it looks so.

---

THE most important precursory sign of diabetic coma is the appearance of acetone in the urine for several days before the attack. Sodium bicarbonate in large doses is of great value in the prodronal stage. The beneficial effect is due likely to the sodium compound neutralizing the lactic and other acids which arise from incomplete oxidation of sugar.—*Exchange.*

---

FRENCH surgeons have recently been using a solution of picric acid for the first treatment of burns. It has been found that the pain which is caused by the burning of the skin can be almost immediately alleviated, or altogether removed, by painting the surface with a strong solution of picric acid.—*Medical Times.*

